# LncRNA MEG3 inhibits rheumatoid arthritis through miR‐141 and inactivation of AKT/mTOR signalling pathway

**DOI:** 10.1111/jcmm.14591

**Published:** 2019-08-14

**Authors:** Guoqing Li, Ying Liu, Fanru Meng, Zhongbin Xia, Xia Wu, Yuxuan Fang, Chunwang Zhang, Yu Zhang, Dan Liu

**Affiliations:** ^1^ Department of Rheumatology, Affiliated Hospital of Yangzhou University Yangzhou University Yangzhou China; ^2^ Clinical Medical College Dalian Medical University Dalian China; ^3^ Department of Pathology, Clinical Medical College Yangzhou University Yangzhou China

**Keywords:** AKT/mTOR, long noncoding RNA, MEG3, miR‐141, rheumatoid arthritis

## Abstract

Rheumatoid arthritis (RA) is a chronic inflammation mediated by autoimmune responses. MEG3, a kind of long noncoding RNA (lncRNA), participates in cell proliferation in cancer tissues. However, the correlation between MEG3 and RA is yet unclear. Therefore, to clarify how MEG3 works in RA, we performed a series of experiments using RA samples. We found that MEG3 was downregulated in the fibroblast‐like synoviocytes of RA patients (RA‐FLS), in comparison with healthy subjects. MEG3 was also down‐regulated evidently in lipopolysaccharide (LPS)‐treated chondrocyte. As part of our experiments, MEG3 was overexpressed in chondrocyte by transfection with lentivirus containing sequences encoding MEG3. In addition, in presence of LPS, reductions were identified not only in the cell proliferation, but also in the generation of interleukin‐23 (IL‐23), which, however were reversed in the lentivirus (containing MEG3‐encoding sequences)‐transfected chondrocytes. Up‐regulated MEG3 resulted in an increase the level of Ki67. Moreover, MEG3 was negatively correlated with miR‐141, and miR‐141 was up‐regulated in LPS‐treated chondrocyte. Inhibitory effects of MEG3 overexpression, mentioned above, were partially abolished by overexpressed miR‐141. Further, animal experiment also showed the inhibitory effect of MEG3 in overexpression on the AKT/mTOR signaling pathway. *In‐vivo*experiments also showed that cell proliferation was facilitated by MEG3 overexpression with inhibited inflammation. In summary, the protective role of MEG3 in RA was proved to be exerted by the increase in the rate of proliferation, which might correlate to the regulatory role of miR‐141 and AKT/mTOR signal pathway, suggesting that MEG3 holds great promise as a therapeutic strategy for RA.

## 1 INTRODUCTION

Rheumatoid arthritis (RA) is a chronic disease mediated by autoimmune responses with manifestations like systemic inflammation.^1^, ^2^ Various studies have shown that genetic and environmental risk factors together contribute to the development of RA.^3^, ^4^, ^5^, ^6^ An increasing number of studies are identifying a correlation between the cytokine networks and the RA pathogenesis.^7^, ^8^ At present, tremendous achievement has been seen in developing effective treatment of RA, in which suppressing the inflammatory responses is the most effect method.^5^, ^9^, ^10^ However, further studies are necessary to discover the mechanism in inflammatory responses of RA.^11^ Long non‐coding RNAs (lncRNAs), transcripts in length longer than 200 nc, show the crucial role in inflammation.^12^, ^13^ For example, in the collagen, antibody induced arthritis mouse model of RA, shikonin can suppress the inflammation by targeting specific lncRNAs,^14^ and in presence of quercetin, the apoptosis of fibroblast‐like synoviocytes (FLS) in RA was also blocked, in which the involvement of lncRNA MALAT1 plays a critical role.^15^, ^16^, ^17^, ^18^, ^19^, ^20^, ^21^, ^22^ In this study, we attempted to discover how MEG3 affects the proliferation and inflammation of LPS‐treated chondrocytes in RA.

## 2 MATERIAL AND METHODS

### 2.1 Cell culture

Chondrocytes were separated from cartilaginous tissues of male SD rats (200‐280 g). (detailed in the Appendix S1).

### 2.2 Lentiviral infection

See Appendix S1.

### 2.3 qRT‐PCR

See Appendix S1.

### 2.4 CCK‐8 assay and crystal violet staining

CCK‐8 assay and crystal violet staining were performed according to manufactures’ instruction (detailed in the Appendix S1).

### 2.5 Western blot analysis

See Appendix S1.

### 2.6 Transfection of mimics

See Appendix S1.

### 2.7 Dual‐luciferase assay

See Appendix S1.

### 2.8 Immunohistochemistry

See Appendix S1.

### 2.9 Flow cytometry

See Appendix S1.

### 2.10 Statistics

GraphPad Prism V was utilized for statistical analysis. The data were expressed as the mean ± standard error of the mean (SEM). All experiments were conducted a minimum of three times. A *P* value of <.05 suggested statistical significance.

## 3 RESULTS

### 3.1 Upregulation of MEG3 in RA

**Figure 1:**
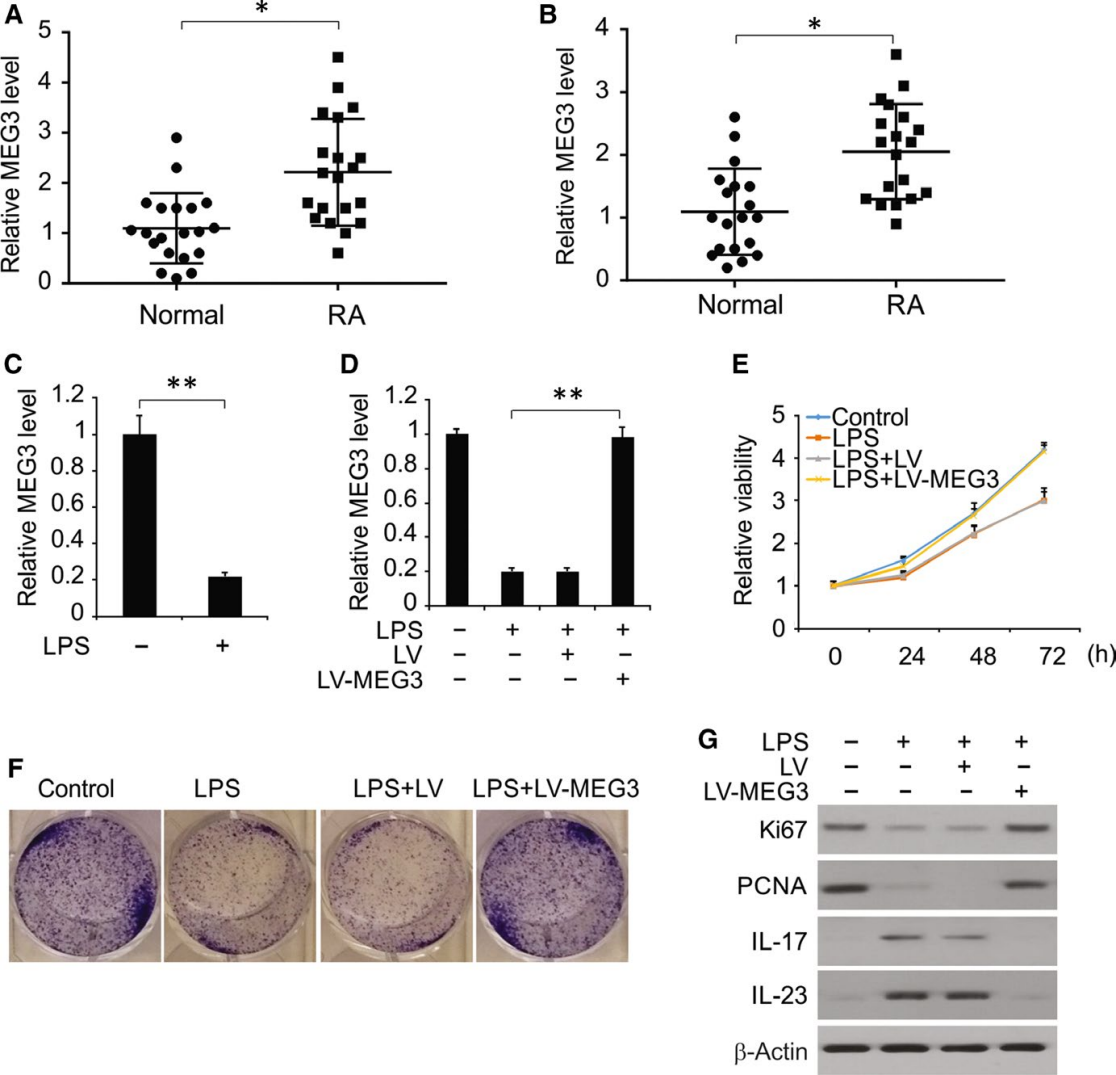
MEG3 affected the proliferative activity and inflammatory responses of LPS‐treated chondrocytes. A, qRT‐PCR results of MEG3 expression in synovial tissues of healthy subjects and RA patients. B, qRT‐PCR results of MEG3 expression in FLS of healthy individuals and RA patients. C, mRNA expression of MEG3 by RT‐PCR. D, mRNA expression of MEG3 in chondrocytes transfected by LV‐MEG3 or negative control, followed by treatment of LPS. E, Proliferative activity of chondrocytes in CCK‐8 assay. F, Crystal violet staining results of chondrocytes. G, Protein expressions of Ki67 and PCNA by Western blotting assay. H, Protein expressions of IL‐17 and IL‐23 by Western blotting assay. *P < .05; **P < .01

To identify the divergence of MEG3 expression in the synovial tissues of healthy and RA subjects, we examined MEG3 expression in these subjects and found significant upregulation of MEG3 expression in the synovial tissue of RA patients (Figure 1A). Moreover, MEG3 expression was up‐regulated in the FLS obtained from the RA synovial tissue (Figure 1B). Thus, upregulation of MEG3 expression is conducive to RA.

**Figure 2:**
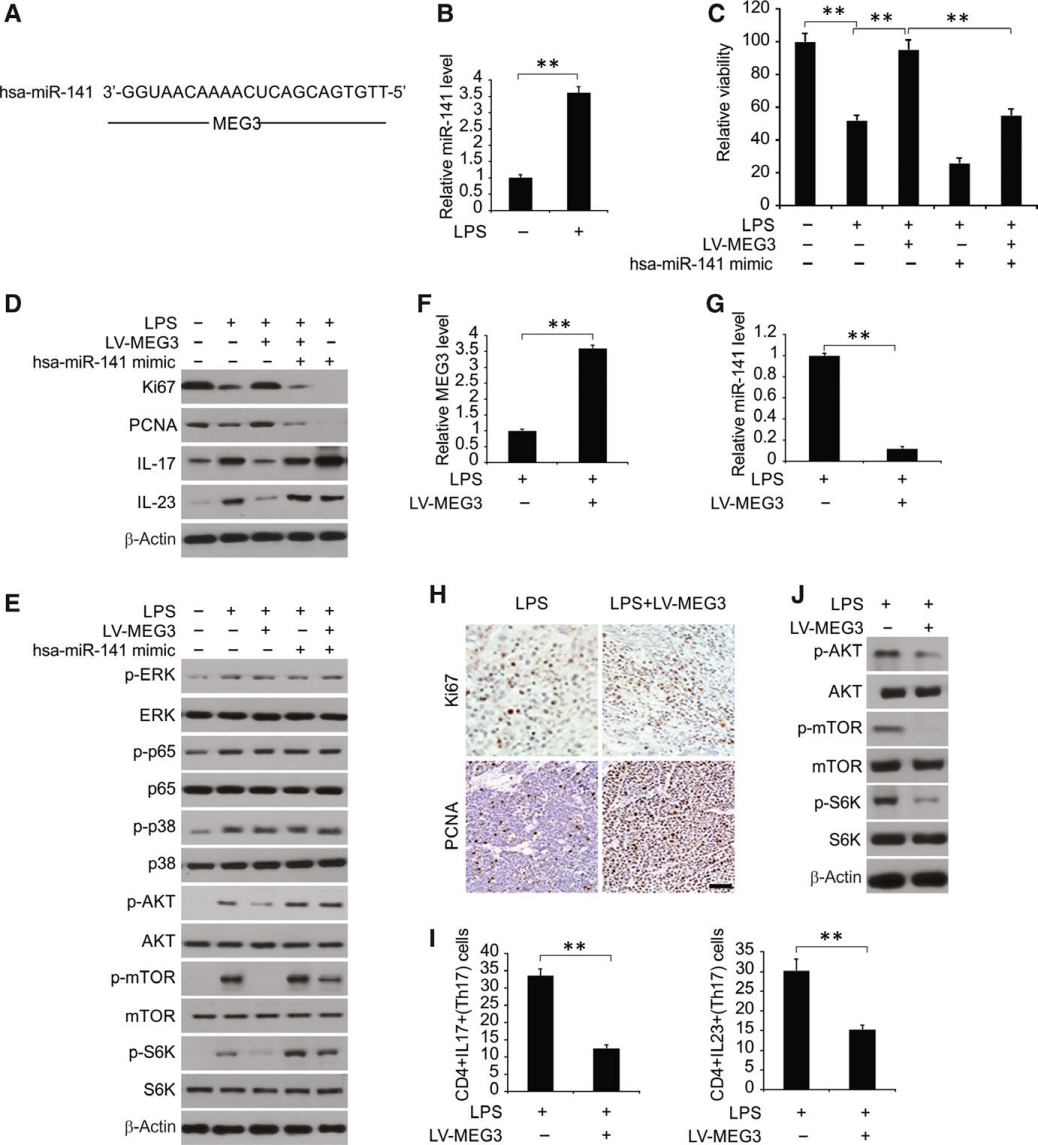
Overexpressing miR‐141 abolished the protective effect of MEG3 on LPS‐treated chondrocytes. A, MEG3 sequences targeted by has‐miR‐141 were analysed by bioinformatics. B, mRNA expression of miR‐141 by RT‐PCR. C, Cell viability assessment using CCK‐8 kit. D, Expressions of Ki67 and PCNA determined by Western blotting assay. E, Protein expressions of IL‐17 and IL‐23 by Western blotting assay. F, Protein expressions of p‐ERK1/2, p‐p65 and p‐P38 were determined by Western blotting. G, Protein expressions of p‐AKT, p‐mTOR and p‐S6K were determined by Western blotting. H, mRNA expression of MEG3 by RT‐PCR. I, mRNA expression of miR‐141 by RT‐PCR. J, Distribution and expressions of Ki67 and PCNA by immunohistochemistry. K, Quantity of CD4^+^ IL‐17^+^ and CD4^+^ IL‐23^+^ cells by FCM. L, Protein expressions of p‐AKT, p‐mTOR and p‐S6K by Western blotting assay. **P < .01

To investigate how MEG3 affects LPS‐treated changes in chondrocytes, we determined the changes in MEG3 level upon LPS treatment. Results showed MEG3 expression was markedly decreased in LPS‐treated chondrocytes (Figure 2A). To find out how MEG3 affects the LPS‐treated changes in proliferation and inflammation of chondrocytes, we overexpressed MEG3 using lentivirus vectors (Figure 1D**)**.As shown in Figure 1E and 1, in comparison with the LPS or Lentiviral (LV) groups, cell viability and proliferation were significantly enhanced in case of MEG3 overexpression. We also found that overexpressed MEG3 up‐regulated Ki67 and PCNA in LPS‐treated chondrocytes (Figure 1G). Moreover, IL‐23 and IL‐17, the pro‐inflammatory cytokines, were down‐regulated upon MEG3 overexpression (Figure 1H). These results indicate that MEG3 protects chondrocytes in the presence of LPS through relieving the inhibitory effect on proliferation as well as down‐regulating the release of pro‐inflammatory cytokines.

### 3.2 Effect of MEG3 on LPS‐treated chondrocytes was reversed by miR‐141 overexpression

It was predicted that there was a possible site on MEG3 sequences that could bind to miR‐141 (Figure 2A), suggesting that they could be correlated. As shown in Figure 2B, miR‐141 was up‐regulated evidently in presence of LPS, in comparison with the control group.

In order to elucidate the role of miR‐141 in the effect exerted by MEG3 on LPS‐treated chondrocytes, a series of transfections were performed using LV‐MEG3, miR‐141 mimic, or the combination, and then treated with LPS. LV‐MEG3 overexpression might protect the cells from LPS‐treated inhibition of proliferation and inflammatory factors production, which was reversed after transfection with miR‐141 mimics (Figure 2C‐E). Thus, under conditions of miR‐141 overexpression, the protective role of MEG3 might be abolished, in part, in LPS‐treated chondrocyte.

### 3.3 MEG3 deactivated miR‐141‐mediated AKT/mTOR signal pathway in LPS‐treated chondrocytes

As shown in Figure 2F and 2, level of p‐ERK1/2, p‐AKT, p‐P38 and p65 were increased in the presence of LPS. In case of MEG3 and miR‐141 overexpression, we found no changes in level of p‐ERK1/2, p‐p65 and p‐P38. However, p‐AKT, p‐mTOR and p‐S6 level were significantly down‐regulated in the LV‐MEG3 group, in comparison with the LPS group, and this down‐regulation was abolished after transfection using miR‐141 mimics. Thus, MEG3/miR‐141 primarily functions by the AKT/mTOR pathway in LPS‐treated signalling.

### 3.4 MEG3 enhanced cell proliferation and suppressed inflammation in vivo

To further clarify the function of MEG3, we carried out in vivo experiments in RA model of rats that were established by infusing LV‐MEG3 subcutaneously. In Figure 2H and 2, MEG3 was up‐regulated after LV‐MEG3 treatment, with a decrease in miR‐141 expression. Ki67 and PCNA levels were augmented in tissue samples with MEG3 overexpression (Figure 2J**)**. Results of Flow cytometry (FCM) revealed that CD4^+^ IL‐23^+^ cells were significantly reduced under the conditions of MEG3 overexpression (Figure 2K). Moreover, MEG3 overexpression down‐regulated the level of p‐AKT in vivo (Figure 2L). Thus, we inferred that MEG3 down‐regulates miR‐141, promotes cell proliferation, reduces Th17 cells and inactivates AKT/mTOR signal pathway in vivo.

## 4 DISCUSSION

With the rapid progress in transcriptome sequencing, different lncRNAs are being identified to be related to different diseases, including RA.^23^, ^24^ Previous study indicated the potential role of MEG3 in RA.^25^ Also, it is important to understand the effect of MEG3 in RA as well as the underlying molecular mechanism. In this study, we found that the suppressed proliferation of chondrocytes that was induced by LPS was reversed by MEG3 overexpression. Furthermore, we found that Ki67 and PCAN were up‐regulated under the conditions of MEG3 overexpression in LPS‐treated chondrocytes. Besides, we also identified a close correlation between inflammatory cytokines and RA. In comparison with the LPS group, overexpressed MEG3 reduced IL‐23 expression remarkably, demonstrating the vital role of MEG3 in the proliferation and inflammatory responses of LPS‐treated chondrocytes.

In this study, we aimed to validate a hypothesis: there is a target relationship between MEG3 and miR‐141, which can be illustrated by bioinformatics‐based prediction. In the presence of LPS, we found that miR‐141 was up‐regulated significantly, in comparison with the control group, which, however, was reversed after MEG3 was overexpressed. Transfection with a miR‐141 mimic significantly reduced cell proliferation and increased the level of IL‐23, prompting that LPS‐induced injuries to chondrocyte may be aggravated in case of miR‐141 overexpression. After chondrocytes were transfected using miR‐141 mimics, the protective effect of MEG3 was abolished on LPS‐treated chondrocytes. Thus, MEG3 can protect the chondrocytes from the LPS‐induced injuries, which is partially due to the inhibition of miR‐141. To discover the role of AKT/mTOR signal pathway in modulating the levels of MEG3 and miR‐141 in LPS‐treated chondrocyte, we studied AKT, p‐AKT, mTOR and p‐mTOR, and found that the AKT/mTOR pathway was activated. In RA models, overexpressed MEG3 increased the expression of Ki67 and PCNA and reduced the number of CD4^+^ IL‐23^+^ cells with down‐regulation in inflammatory cytokines, which were in agreement with the results of in vitro experiments. Thus, MEG3 promotes in vitro and in vivo proliferation of cells and suppresses inflammation.

## CONFLICT OF INTEREST

The authors declare that they have no competing interests.

## AUTHOR CONTRIBUTIONS

In this work, Guoqing Li and Dan Liu conceived the study and designed the experiments. Ying Liu, Fanru Meng, Zhongbin Xia and Xia Wu contributed to the data collection, Yuxuan Fang, Chunwang Zhang and Yu Zhang performed the data analysis and interpreted the results. Guoqing Li wrote the manuscript; Guoqing Li and Dan Liu contributed to the critical revision of article. All authors read and approved the final manuscript.

## Data Availability

All data generated or analysed during this study are included in this published article.
