# Supplementary material for: LncRNA MEG3 inhibits rheumatoid arthritis through miR‐141 and inactivation of AKT/mTOR signalling pathway
Source: J Cell Mol Med. 2019 Aug 14;23(10):7116–20. doi: 10.1111/jcmm.14591 (PMC6787440; doi:10.1111/jcmm.14591)
Supplement: Supplementary file 1 [file JCMM-23-7116-s001.docx]

**Material and methods**

**Cell culture**

Chondrocytes were separated from cartilaginous tissues of male SD rats (200-280 g). Following three washes in Dulbecco’s Modified Eagle Medium (DMEM, Gibco), cartilaginous tissues were cut into pieces for digestion using the corresponding reagent overnight at 37°C. Then, cell suspension was prepared in DMEM regularly. At the time of confluence, cells were collected for culture to confluence again.

After treatment with LPS (Sigma, St. Louis, MO, USA) in varying concentrations in chondrocytes, we found that LPS affected cell viability and reduce MEG3 expression in a concentration-dependent manner, in which the maximal effect emerged at the concentration of 10 µg/mL. Thus, 10 μg/mL was used as the optimal concentration of LPS in further experiments.

**Lentiviral (LV) infection**

LV-MEG3 (carrying coding sequences of MEG3), LV (empty LV), or polybrene (Genechem, Shanghai, China) were used for infection of chondrocytes for 48 h, followed by qRT-PCR to evaluate the infection efficiency.

**qRT-PCR**

In presence of TRIzol reagent (Sigma), the cytoplasmic and nuclear RNA were extracted according to the standard procedure for reverse transcription of miRNA by First-Strand Synthesis Kit (Clontech, Mountain View, CA, USA), with U6 as the internal reference. Total RNA was also used for the reverse transcription of lncRNAs by using the First-Strand cDNA Synthesis Kit (Thermo Scientific, Pittsburgh, PA, USA), with β-actin as the internal reference.

**CCK-8 assay and crystal violet staining**

For measurement of cell proliferation, we utilized the CCK-8 kit (Abcam, Shanghai, China) to perform CCK-8 assay: Cells were seeded on a 96-well plate and incubated in CCK-8 solution for 4 hours at 37°C, followed by measurement of the absorbance at wavelength of 450 nm. To evaluate the viability of cells, we stained the cells using crystal violet as previously described.

**Western blot analysis**

Total proteins were isolated from the chondrocyte lysate prepared in the phenylmethane sulfonyl fluoride-supplemented radio-immunoprecipitation assay (RIPA) solution, and protein concentration was measured using bicinchoninic acid (BCA) assay. Samples with similar protein content were subjected to sodium dodecyl sulfate-polyacrylamide gel electrophoresis (SDS-PAGE), and the proteins were electro-transferred onto the polyvinylidene fluoride (PVDF, Sigma) membrane, followed by blocking in 5% skimmed milk. The membrane-bound proteins were probed using primary antibodies at 4°C overnight, followed by horseradish peroxidase (HRP)-labeled secondary antibody for 2 hours at room temperature. Immunoblot visualization was performed with Pierce ECL reagent (Thermo Fisher Scientific), followed by quantification with Image J. β-actin was used as the internal reference.

**Transfection of mimics**

MiR-141 mimic (Life Technologies, Carlsbad, CA, USA) was used for transfecting the chondrocytes in presence of [Lipofectamine](http://www.sciencedirect.com/topics/pharmacology-toxicology-and-pharmaceutical-science/lipofectamine) 2000 (Invitrogen, Waltham, MA, USA). Two days later, we evaluated the transfection efficiency through qRT-PCR.

**Dual-luciferase assay**

Wild-type MEG3 sequence (MEG3-WT), and the sequence containing mutations (MEG3-MUT) that was designed to bind to miR-141, were delivered in the pGL3 vector. In brief, chondrocytes were firstly seeded on a 96-well plate, and then transfected simultaneously by miR-141 mimic and pGL3 vectors above for 48 hours, followed by dual-luciferase assay in strict accordance with the manufacturer’s instruction (Promega).

**Immunohistochemistry**

The Rat model experiment was approved by Yangzhou University. From the RA models of rat, the cartilage samples were collected, fixed in paraformaldehyde, and embedded in the paraffin. Tissues in paraffin were sliced into sections in thickness of 5 μm, and after regular dewaxing and hydrating, sections were placed in 30% peroxide for blocking the activity of peroxidase, followed by antigen retrieval in citrate buffer. Thereafter, sections were incubated with anti-Ki67 or -PCNA antibodies overnight at 4°C, followed by HRP-conjugated secondary antibody. Color of sections were developed using the DAB (R&D Systems, Minneapolis, MN, USA).

**Flow cytometry (FCM)**

[In](http://www.sciencedirect.com/topics/immunology-and-microbiology/peripheral-blood-mononuclear-cell) this experiment, peripheral blood mononuclear cells (PBMCs) were collected from the RA rats and isolated for following determination. PBMCs were then incubated in presence of FITC-labeled anti-CD4 antibody and PE-labeled anti-IL-17 or anti-IL-23 antibody for FCM, with CELLQuest software to analyze the results.

**Statistics**

GraphPad Prism V was utilized for statistical analysis. The data were expressed as the mean ± standard error of the mean (SEM). All experiments were conducted a minimum of three times. A P value of <0.05 suggested statistical significance.
